# Bioleaching of Gold from Sulfidic Gold Ore Concentrate and Electronic Waste by *Roseovarius tolerans* and *Roseovarius mucosus*

**DOI:** 10.3390/microorganisms8111783

**Published:** 2020-11-14

**Authors:** Kanjana Kudpeng, Tsing Bohu, Christina Morris, Paitip Thiravetyan, Anna H. Kaksonen

**Affiliations:** 1 Commonwealth Scientific and Industrial Research Organisation (CSIRO) Land and Water, 147 Underwood Avenue, Floreat, WA 6014, Australia; t_t_2535@hotmail.com (K.K.); christina.morris@csiro.au (C.M.); 2 School of Bioresources and Technology, King Mongkut’s University of Technology Thonburi, Bangkok 10150, Thailand; paitip.thi@kmutt.ac.th; 3 CSIRO Mineral Resources, Australian Resources and Research Centre, Kensington, WA 6151, Australia; qing.hu@csiro.au; 4 School of Biomedical Sciences, University of Western Australia, Crawley, WA 6009, Australia

**Keywords:** bioleaching, concentrate, electronic waste, gold, iodide oxidising bacteria, printed circuit board, *Roseovarius mucosus*, *Roseovarius tolerans*, sulfidic ore concentrate

## Abstract

Gold bioleaching mediated by iodide oxidizing bacteria (IOB) has been proposed as a sustainable alternative to conventional technologies such as cyanidation. This study evaluated the ability of two IOB sourced from a commercial culture collection, *Roseovarius* (*R.*) *tolerans* DSM 11457^T^ and *R. mucosus* DSM 17069^T^, to bioleach gold from electronic waste (e-waste) (1030 ppm gold) and sulfidic gold ore concentrate (45 ppm gold) using one-step, two-step and spent medium leaching at 1% pulp density over 10 days. Two-step bioleaching of ore concentrate resulted in the highest gold leaching yields (approximately ~100% and 34% for *R. tolerans* and *R. mucosus*, respectively), followed by spent medium leaching and one-step leaching. The yields remained low for e-waste with both strains (maximum 0.93% and 1.6% for *R. tolerans* and *R. mucosus*, respectively) and decreased over time, likely due to the instability of the solubilized gold at relatively low redox potentials (<300 mV vs. Ag/AgCl). Another limiting factor may be the partial inhibition of bacterial growth in the presence of the ore concentrate and e-waste. Therefore, future studies should evaluate the pre-treatment of the ore concentrate and e-waste to remove inhibitory and oxidant consuming compounds before bioleaching with IOB to optimize leaching yields.

## 1. Introduction

Bioleaching refers to the solubilization of target metals by microorganisms from materials such as minerals and wastes [1,2,3,4]. Considering the relatively low environmental and operating cost of bioleaching, numerous studies have explored the use of various microorganisms for biosolubilization [5]. Redoxolysis, acidolysis, and complexolysis are the main mechanisms to drive bioleaching. Chemolithoautotrophs such as *Acidithiobacillus* (*A*.) *ferrooxidans* and *A. thiooxidans* have been used to solubilize base metals, especially copper [6,7,8]. These species oxidize ferrous iron to ferric iron and/or reduced sulfur compounds to sulfuric acid, which leach metals from minerals and wastes via redoxolysis and acidolysis, respectively [6,9,10]. Heterothophic fungi, such as *Aspergillus niger* and *Penicillium simplicissimum* have also been employed for bioleaching metals from electronic waste (e-waste) [1,11,12]. Fungal bioleaching occurs through acidolysis and complexolysis, facilitated by the production of organic acids such as citric, oxalic and gluconic acid [6]. Some fungi have additionally been shown to oxidize gold [13].

Recently, the application of iodide oxidizing bacteria (IOB) for bioleaching gold was proposed [10] and demonstrated for gold-containing sulfide ore [14]. A heterotrophic bacterium “*Pseudomonas iodooxidans*” has been reported to oxidize I^−^ to I_2_ with hydrogen peroxide (H_2_O_2_) as an electron acceptor (Reaction 1) [15,16,17,18]:

H_2_O_2_ + 2I^−^ + 2H^+^ → I_2_ + 2H_2_O (1)

Another strain, *Roseovarius* (*R*.) sp., can oxidise I^−^ to I_2_ with oxygen as the electron acceptor (Reaction 2) [18,19,20,21]:

4I^−^ + O_2_ + 4H^+^ → 2I_2_ + 2H_2_O (2)

Iodide (I^−^) reacts chemically with iodine (I_2_) to form in triiodide (I_3_^−^) according to Reaction (3):

I^−^ + I_2_ → I_3_^−^ (3)

Gold can be solubilised according to reactions (4) and (5):

2Au + I_3_^−^ + I^−^ → 2[AuI_2_]^−^ (4)

2Au + 3I_3_^−^ → 2[AuI_4_]^−^ + I^−^ (5)

E-waste generation is rapidly increasing globally [22]. The composition of e-waste is very heterogenous, and includes iron, non-ferrous metals, plastics and other constituents (e.g., rubber, concrete and ceramics) [22]. In terms of metallic components, e-waste contains precious metals (Ag, Au and Pt), base metals (Al, Co, Cu, Ni, Zn and Fe) and others (e.g., Be, Cd, Cr, Hg, Pb, Sb, Sn and Ti). If improperly managed, e-waste can cause toxicity to humans and the environment. On the other hand, e-waste contains high amounts of precious metals, the extraction and recovery of which is warranted. Bioleaching of e-waste has been explored as an alternative to chemical leaching, especially using cyanide forming microorganisms [23,24,25]. However, due to the high toxicity of cyanide, more environmentally friendly biolixiviants should be explored. To the best of our knowledge, the use of IOB for bioleaching gold from e-waste and sulfidic gold ore concentrate has not been previously explored. Therefore, the objectives of this study were (1) to evaluate the ability of IOB, *R. tolerans* and *R. mucosus*, to bioleach gold from e-waste and sulfidic gold ore concentrate, and (2) to evaluate the effect of various leaching approaches (one-step, two-step and spent medium bioleaching) on gold extraction.

## 2. Materials and Methods

### 2.1. Preparation and Analysis Of Sulfide Ore Concentrate and e-Waste

Sulfidic gold ore concentrate was sourced from a gold mine in Western Australia and had a particle size with a P80 (i.e., 80% of mass passing) of 120 µm. The gold ore concentrate was sterilized by autoclaving before leaching experiments (121 °C for 20 min). Shredded e-waste (high-grade printed circuit boards) was sourced from Total Green Recycling in Perth, Western Australia. The shredded e-waste was pulverized with Essa^®^ LM5 mill (FMSmidth, Denmark) at Nagrom, Western Australia.

### 2.2. Iodide Oxidising Bacteria (IOB) Culture Conditions

The IOB species used in this study, *R. tolerans* DSM 11457^T^ and *R. mucosus* DSM 17069^T^, were selected based on previous report by [14] and ordered from the German Collection of Microorganisms and Cell Cultures GmbH (DSMZ, Braunschweig, Germany). Both strains were cultured in 37.4 g·L^−1^ of Difco™ Marine Broth 2216 that contained (g·L^−1^): 5 peptone, 1 yeast extract, 0.1 C_6_H_5_FeO_7_, 19.45 NaCl, 5.9 MgCl_2_, 3.24 MgSO_4_, 1.8 CaCl_2_, 0.55 KCl, 0.16 NaHCO_3_, 0.08 KBr and (mg·L^−1^): 34 SrCl_3_, 22 H_3_BO_3_, 4 NaSiO_3_, 2.4 NaF, 1.6 NH_4_NO_3_ and 8 Na_2_HPO_4_. The cultures were incubated under aerobic condition at 100 rpm and 30 °C for two days. The initial cell number for the bioleaching experiments was adjusted to 3 × 10^6^ cell·mL^−1^ according to previous study [14].

### 2.3. Bioleaching Experiments

The culture medium for gold bioleaching by IOB contained 18.7 g·L^−1^ of Difco™ Marine Broth 2216 and 10.9 g·L^−1^ of potassium iodide (KI) (pH 7.2). A 50 mL liquid volume was used in a 250 mL flask for leaching experiments. The culture medium was sterilized at 121 °C for 20 min. Thereafter, 10% of inoculum (5 mL) was added into 45 mL of culture medium followed by 0.5 g of milled e-waste or gold ore concentrate for a one-step bioleaching at 1% pulp density. For two-step bioleaching, the 50 mL culture was incubated for 3 days before adding 0.5 g e-waste or gold ore concentrate for 1% pulp density. For spent medium bioleaching, cells from a 3-day culture were removed by 0.2 μm syringe filtration (Millex, Merck Millipore, Ireland). Thereafter, 50 mL of filtrate was transferred to a new 250 mL flask and 0.5 g e-waste or gold ore concentrate was added to achieve 1% pulp density. Spent medium condition was used to study the ability of metabolites from *Roseovarius* spp. to leach gold. The chemical ore and e-waste controls had only ore concentrate or e-waste in the medium, respectively, without bacterial inoculum. The medium control had medium only without ore concentrate, e-waste or bacteria. The biological bacteria control had the medium inoculated with bacteria, but no ore concentrate or e-waste. Each condition was tested in triplicate. All leaching experiments were conducted for 10 days at 30 °C, in a shaker (Innova44, Eppendorf, Enfield, CT, USA) at 100 rpm. Samples were taken at days 0, 5 and 10 for the analysis of soluble gold, pH, redox potential, triiodide concentration and bacterial cell numbers.

### 2.4. Analytical Methods

The elemental compositions of the gold ore concentrate and e-waste were analysed at LabWest, Western Australia. Platinum group metals were determined by a PGM fire assay followed by aqua regia digestion and analysis by inductively coupled plasma optical emission spectrometry (ICP-OES). Other metals were analysed by microwave assisted digestion with a mixture of HF, HCl and HNO_3_, followed by analysis using a combination of ICP-OES and ICP mass spectrometry (MS).

For analysing soluble gold, 4 mL aliquots of samples filtered through 0.2 μm syringe filters were acidified with 0.1 mL of 7 M nitric acid and 7.5 mL of 10% hydrochloric acid and diluted with 3.4 mL ultrapure water to a final volume of 15 mL. Soluble gold concentrations were measured by ICP-MS at The Commonwealth Scientific and Industrial Research Organisation (CSIRO) Mineral Resources Waterford laboratory, Western Australia. The pH and redox potential of filtrate solution were measured at room temperature using TPS smart CHEM-Lab meter with a TPS pH probe (EPBUFN-121207) and a TPS redox probe (EOREFN-121262) with Ag/AgCl reference.

Triiodide concentration was measured using a spectrometric method described by [26]. Triiodide standards were prepared by dissolving potassium iodide (KI) and iodine to obtain 100 mM iodide and 7.88 mM iodine solutions, respectively. Then, triiodide was formed by mixing iodine solution and potassium iodide solution (Reaction 3). The standard was prepared with 0.5 mM of potassium iodide and 0, 0.005, 0.01, 0.02, 0.04, 0.06, 0.08, and 0.10 mM of iodine. The absorbance was measured at 351 nm using a microplate reader (Varioskan^TM^ LUX, Thermo Scientific, Vantaa, Uusimaa, Finland). Numbers of suspended cells were counted by phase contrast microscopy (Leica DM4000 B, Germany) using Helber bacterial counting chamber.

### 2.5. Calculations and Statistical Analysis

The gold leaching yields were calculated using Equations (6)–(10). The mass of the dissolved gold collected during sampling (*MR_i_*, mg) was estimated from Equation (6):

(6) MRi=Ci×SVi

where *C_i_* is the dissolved gold concentration in the sample (mg·L^−1^) and *SV_i_* is the subsample volume (L). Cumulative mass of dissolved gold removed in samples (CMR, mg) was calculated using Equation (7):

(7) $$CMR=\overset{n}{\underset{i=0}{\sum }}MRi$$

The mass of gold dissolved in the remaining leachate in the flask (SM, mg) was estimated using Equation (8):

(8) SM=C×V

where *C* is the dissolved gold concentration in the leachate (mg·L^−1^) and *V* is the remaining volume of leachate in the flask (L). The total mass of dissolved gold (TL, mg) was calculated using Equation (9):

(9) TL=CMR+SM

The leaching yield (Y, %) was calculated using Equation (10):

(10) $$Y=\;\frac{\left(TL\times \;100\right)}{Original\;gold\;mass}$$

where *Original gold mass* refers to the mass of gold (mg) in the ore concentrate or e-waste used for leaching. Averages and standard deviations of gold leaching yields were determined for each time point.

Two- and one-way analyses of variance (ANOVA) were used to determine the statistical significance of the differences detected between bioleaching conditions. Statistical tests were conducted in Microsoft Excel 365 ProPlus and *p*-values < 0.05 were considered significant.

## 3. Results

### 3.1. Elemental Composition of Sulfide Ore Concentrate and e-Waste

The elemental composition of the gold ore concentrate and e-waste were as shown in Table 1 and Table 2, respectively. Gold contents in the gold ore concentrate and e-waste were approximately 45 ppm and 1030 ppm, respectively.

### 3.2. Gold Bioleaching from Sulfide Ore Concentrate and e-Waste by R. tolerans and R. mucosus

**Figure 1:**
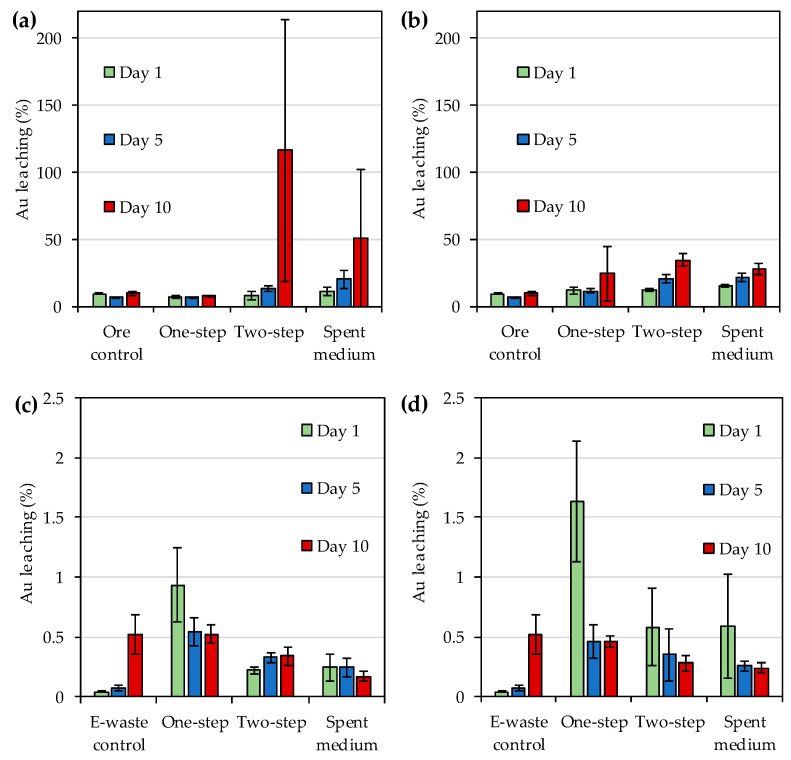
(**a**) Gold bioleaching from sulfidic gold ore concentrate by *Roseovarius* (*R*.) *tolerans*; (**b**) Gold bioleaching from sulfidic gold ore concentrate by *R. mucosus*; (**c**) Gold bioleaching from e-waste by *Roseovarius* (*R*.) *tolerans*; (**d**) Gold bioleaching from e-waste by *R. mucosus*. Ore control and e-waste control had either ore concentrate or e-waste in medium, respectively without bacterial inoculum. In one-step bioleaching, sulfidic gold ore concentrate or e-waste were added at the same time as inoculum. For two-step bioleaching, the cultures were pre-cultivated for 3 days before the addition of sulfidic gold ore concentrate or e-waste. Spent medium bioleaching was conducted with filtered culture medium after 3 days of incubation. Note different vertical scales for gold leaching from ore concentrate (**a** and **b**) and e-waste (**c** and **d**).

Gold leaching yields from gold ore concentrate and e-waste by *R. tolerans* and *R. mucosus* were as shown in Figure 1 and *p*-values from two- and one-way ANOVA were as shown in Table 3 and Table 4, respectively. When bioleaching gold ore concentrate, for both species two-step bioleaching resulted in the highest gold yields, followed by spent medium leaching and one-step leaching (Figure 1a,b). The yields increased over time and on day 10 were approximately ~100% and 34% for two-step leaching and 51% and 28% for spent medium leaching for *R. tolerans* and *R. mucosus*, respectively. However, standard deviations between replicates were high for some leaching methods for day 10 sampling. As a result, the impact of bacterial species or leaching method (excluding uninoculated ore control) on gold leaching from ore concentrate was not significant for day 10, although *p*-values indicated significant impact in two-way ANOVA for days 1 and 5 (Table 3). In one-way ANOVA for evaluating the effect of leaching method (including uninoculated ore control) separately for each bacterial species, significant *p*-values were recorded for *R. tolerans* for day 5 and *R. mucosus* for days 1 and 5 (Table 4).

When bioleaching gold from e-waste the yields were much lower for both strains as compared to the yields obtained for the gold ore concentrate (Figure 1). The highest yields for e-waste were obtained with one-step leaching on day 1, namely 0.93% and 1.6% for *R. tolerans* and *R. mucosus*, respectively, while the day 1 yield in uninoculated e-waste control was 0.042% (Figure 1c,d). Surprisingly, the yields decreased over time for one-step and spent medium bioleaching for both strains, and for two-step leaching with *R. mucosus*, whereas the yield increased somewhat over time in two-step leaching with *R. tolerans* and in uninoculated e-waste controls. However, by day 10, the leaching yields with microorganisms were similar or lower than those obtained with uninoculated e-waste control (Figure 1c,d). In two-way ANOVA, the impact of bacterial species on gold leaching from e-waste was significant only on day 1 and the impact of leaching method (excluding uninoculated ore control) was significant for days 1 and 5 (Table 3). In one-way ANOVA for evaluating the effect of leaching method (including uninoculated ore control) separately for each bacterial species, significant *p*-values were recorded for both *R. tolerans* and *R. mucosus* for days 1, 5 and 10 (Table 4). However, it is to be noted that while bioleaching resulted in higher leaching than in e-waste control for days 1 and 5, the uninoculated control showed higher leaching than most bioleaching methods on day 10 (Figure 1c,d).

### 3.3. Triiodide Concentrations during Gold Bioleaching

**Figure 2:**
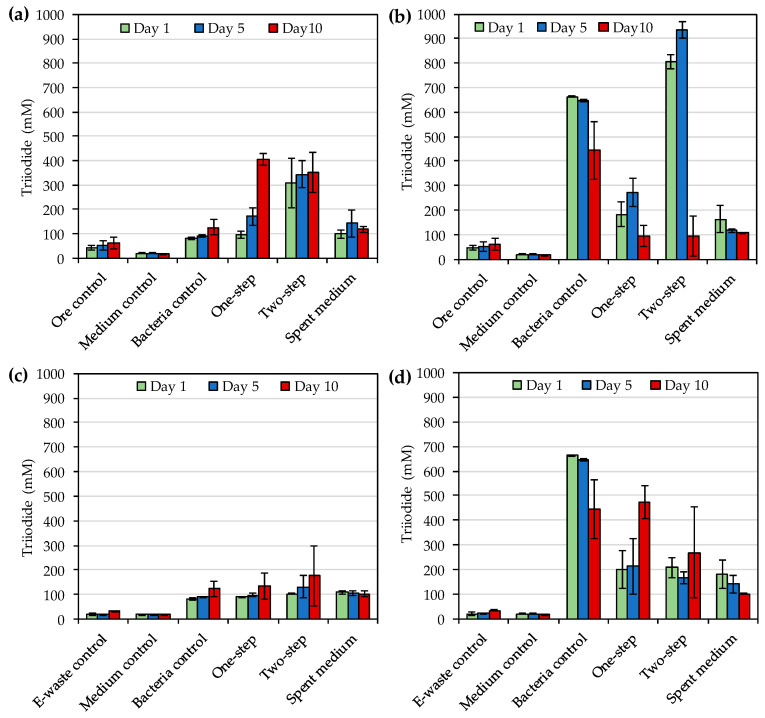
(**a**) Triiodide concentrations during bioleaching of gold from sulfidic gold ore concentrate by *Roseovarius* (*R*.) *tolerans*; (**b**) Triiodide concentrations during bioleaching of gold from sulfidic gold ore concentrate by *R. mucosus*; (**c**) Triiodide concentrations during bioleaching of gold from e-waste by *R*. *tolerans*; (**d**) Triiodide concentrations during bioleaching of gold from e-waste by *R. mucosus*. Ore control and e-waste control had either ore concentrate or e-waste in medium, respectively without bacterial inoculum. Medium control had medium only, and bacteria control had inoculated medium without ore concentrate or e-waste. In one-step bioleaching, sulfidic gold ore concentrate or e-waste were added at the same time as inoculum. For two-step bioleaching, the cultures were pre-cultivated for 3 days before the addition of sulfidic gold ore concentrate or e-waste. Spent medium bioleaching was conducted with filtered culture medium after 3 days of incubation.

The concentrations of triiodide during the bioleaching of gold from ore concentrate and e-waste with *R. tolerans* and *R. mucosus* were as shown in Figure 2 and *p*-values from two- and one-way ANOVA were as shown in Table 5 and Table 6, respectively. For bioleaching of gold ore concentrate with *R. tolerans*, triiodide concentrations were higher in one-step and two-step leaching than in the bacterial control (bacteria without ore concentrate) and spent medium leaching as shown in Figure 2a. On the other hand, for bioleaching gold ore concentrate with *R. mucosus*, two-step leaching resulted in the highest triiodide concentrations, followed by bacteria control (Figure 2b). The highest triiodide concentration for *R. mucosus* was detected on day 5 with two-step leaching (934 mg·L^−1^). This was more than double the highest triiodide concentration detected for *R. tolerans* on day 10 with one-step leaching (406 mg·L^−1^). The impact of bacterial species on triiodide concentration during gold leaching from ore concentrate was significant for days 1 and 5, but not on day 10, whereas *p*-values indicated significant impact of leaching method (excluding uninoculated ore control) for all sampling times in two-way ANOVA (Table 5). In one-way ANOVA for evaluating the effect of leaching method (including uninoculated ore control, medium control and bacteria control) on triiodide concentrations during ore concentrate bioleaching separately for each bacterial species, significant *p*-values were recorded for *R. tolerans* and *R. mucosus* for all sampling days (Table 6).

When bioleaching e-waste, the triiodide concentrations were notably lower for both strains than when bioleaching gold ore concentrate (Figure 2c,d). The highest detected concentrations in the presence of e-waste were 472 mg·L^−1^ for *R. mucosus* on day 10 for one-step leaching and 176 mg·L^−1^ for *R. tolerans* on day 10 for two-step leaching (Figure 2c,d). In two-way ANOVA, the impact of bacterial species on gold leaching from e-waste was significant on all sampling days, but the impact of leaching method (excluding uninoculated ore control) was significant only for day 10 (Table 5). In one-way ANOVA for evaluating the effect of leaching method (including uninoculated ore control, media control and bacterium control) on triiodide concentrations during e-waste bioleaching separately for each bacterial species, significant *p*-values were recorded for both *R. tolerans* and *R. mucosus* for all sampling days (Table 6).

### 3.4. Cell Numbers during Gold Bioleaching

**Figure 3:**
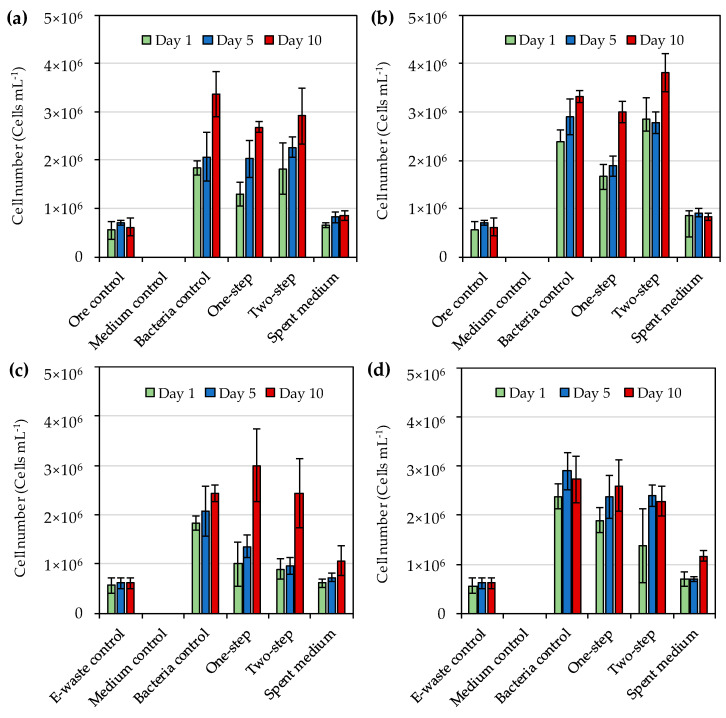
(**a**) Cell numbers during bioleaching of gold from sulfidic gold ore concentrate by *Roseovarius* (*R*.) *tolerans*; (**b**) Cell numbers during bioleaching of gold from sulfidic gold ore concentrate by *R. mucosus*; (**c**) Cell numbers during bioleaching of gold from e-waste by *R*. *tolerans*; (**d**) Cell numbers during bioleaching of gold from e-waste by *R. mucosus*. Ore control and e-waste control had either ore concentrate or e-waste in medium, respectively without bacterial inoculum. Medium control had medium only, and bacteria control had inoculated medium without concentrate or e-waste. In one-step bioleaching, ore concentrate or e-waste were added at the same time as inoculum. For two-step bioleaching, the cultures were pre-cultivated for 3 days before the addition of ore concentrate or e-waste. Spent medium bioleaching was conducted with filtered culture medium after 3 days of incubation.

The initial cell number at the start of bioleaching was set as 3 × 10^6^ cell·mL^−1^. Interestingly, after the addition of sulfidic gold ore concentrate and e-waste, cell numbers temporarily decreased, but then increased over time for both strains, in the presence of both gold ore concentrate and e-waste (Figure 3) However, the cell numbers remained in the same order of magnitude as at the start of the leaching. Moreover, the final cell numbers for one and two-step leaching were similar to those in bacterial control (without ore concentrate or e-waste). Cell numbers in medium control remained below detection limits and those in ore and e-waste controls and spent medium remained below 1 × 10^6^ cells·mL^−1^ apart from day 10 for spent medium leaching of e-waste when the numbers reached slightly over 1 × 10^6^ cells·mL^−1^ (Figure 3a–d). The cells in the spent medium flasks likely originated from the ore concentrate and e-waste and were unlikely to oxidize iodide as no notable increase in triiodide concentration was detected during the leaching in the spent medium flasks.

### 3.5. Redox Potential and pH during Gold Bioleaching

**Figure 4:**
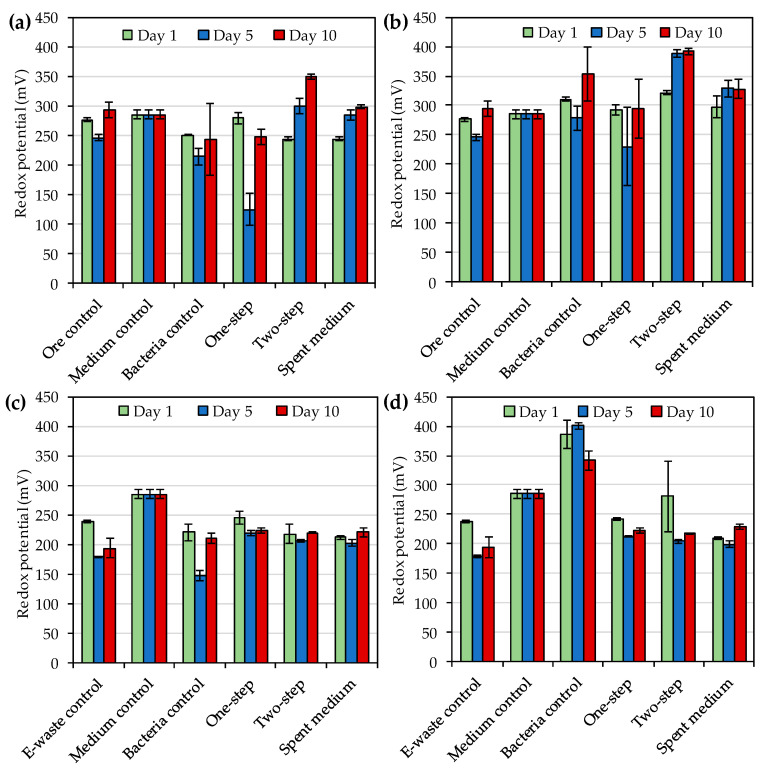
(**a**) Redox potential (vs. Ag/AgCl reference) during bioleaching of gold from sulfidic gold ore concentrate by *Roseovarius* (*R*.) *tolerans*; (**b**) Redox potential during bioleaching of gold from sulfidic gold ore concentrate by *R. mucosus*; (**c**) Redox potential during bioleaching of gold from e-waste by *R*. *tolerans*; (**d**) Redox potential during bioleaching of gold from e-waste by *R. mucosus*. Ore control and e-waste control had either ore concentrate or e-waste in medium, respectively without bacterial inoculum. Medium control had medium only, and bacteria control had inoculated medium without concentrate or e-waste. In one-step bioleaching, ore concentrate or e-waste were added at the same time as inoculum. For two-step bioleaching, the cultures were pre-cultivated for 3 days before the addition of ore concentrate or e-waste. Spent medium bioleaching was conducted with filtered culture medium after 3 days of incubation.

**Figure 5:**
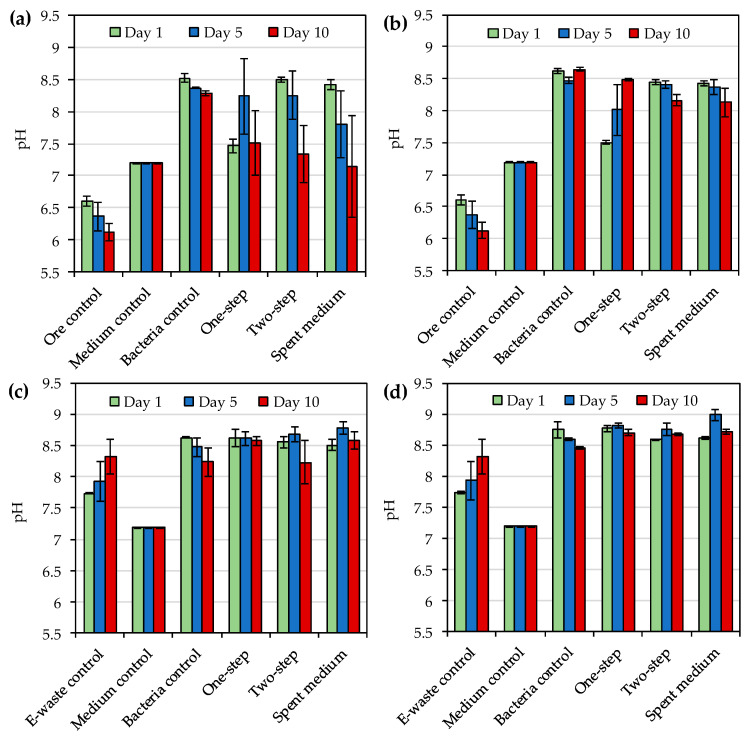
(**a**) Solution pH during bioleaching of gold from sulfidic gold ore concentrate by *Roseovarius* (*R*.) *tolerans*; (**b**) Solution pH during bioleaching of gold from sulfidic gold ore concentrate by *R. mucosus*; (**c**) Solution pH during bioleaching of gold from e-waste by *R*. *tolerans*; (**d**) Solution pH during bioleaching of gold from e-waste by *R. mucosus*. Ore control and e-waste control had either ore concentrate or e-waste in medium, respectively without bacterial inoculum. Medium control had medium only, and bacteria control had inoculated medium without concentrate or e-waste. In one-step bioleaching, ore concentrate or e-waste were added at the same time as inoculum. For two-step bioleaching, the cultures were pre-cultivated for 3 days before the addition of ore concentrate or e-waste. Spent medium bioleaching was conducted with filtered culture medium after 3 days of incubation.

The redox potentials and solution pH values during the bioleaching of gold ore concentrate and e-waste were as shown in Figure 4 and Figure 5. *R. tolerans* decreased redox potential somewhat as compared to media control, whereas *R. mucosus* slightly increased redox potential (Figure 4). Redox potential increased over time for two-step and spent medium bioleaching of ore concentrate with both strains, whereas similar trend was not detected for one-step bioleaching of the concentrate or for bioleaching of e-waste with any of the three approaches. The highest redox potentials for ore concentrate bioleaching were detected on day 10 with two-step bioleaching for both *R. tolerans* (350 mV vs. Ag/AgCl) and *R. mucosus* (392 mV vs. Ag/AgCl) (Figure 4a,b). For *R. mucosus* the presence of e-waste decreased redox potential as compared to bacterial control, whereas for *R. tolerans* the effect of e-waste on redox potential was not notable as compared to bacteria control (Figure 4c,d).

The addition of ore concentrate decreased solution pH as compared to medium control (Figure 5a,b) whereas e-waste increased solution pH (Figure 5c,d). The presence of bacteria and/or their metabolites increased pH as compared to media control both in the absence and presence of ore concentrate and e-waste, especially on days 1 and 5. However, for *R. tolerans* the solution pH decreased somewhat by day 10, especially in the presence of the ore concentrate.

## 4. Discussion

The application of IOB for bioleaching gold has been previously proposed as a sustainable alternative to cyanide [10] and, the use of IOB isolated from iodide-rich brine waters has been explored for leaching gold from gold-containing ore [14,21]. This study evaluated the ability of IOB sourced from a commercial culture collection, *R. tolerans* DSM 11457^T^ and *R. mucosus* DSM 17069^T^, to bioleach gold from e-waste and sulfidic gold ore concentrate. *R. tolerans* DSM 11457^T^ was originally isolated from a hypersaline lake in East Antarctica [27] whereas *R. mucosus* DSM 17069^T^ was isolated from a marine dinoflagellate [28]. Neither of the strains was described as an IOB in the original species descriptions, but were selected for this study based on other reports on the members of *Roseovarius* genus being able to oxidize iodide [14,18,21]. While previous studies have evaluated the use of strains of *Roseovarius* genus to leach gold from ore using one-step leaching, this study explored gold bioleaching with *R. tolerans* DSM 11457^T^ and *R. mucosus* DSM 17069^T^ from gold ore concentrate and e-waste using one-step, two-step and spent medium bioleaching. Both species were able to bioleach gold from ore concentrate, and two-step bioleaching resulted in the highest gold yields, followed by spent medium leaching and one-step leaching (Figure 1a,b). However, the yields were much lower for e-waste with both strains as compared to the yields obtained for the gold ore concentrate (Figure 1). While bioleaching yields generally increased over time for ore concentrate, declining trend in yields was recorded for e-waste during the experiment. A comparison of maximum bioleaching yields achieved in this study and selected previous studies is shown in Table 7. The maximum gold yield detected for *R. tolerans* with gold concentrate in this study was similar to that reported by Khaing et al. [14,21] for other *R. tolerans* strains leaching sulfide ore. However, the maximum yield obtained with *R. mucosus* for ore concentrate was lower than that previously reported for strains of *Roseovarius* genus. The bioleaching yields for e-waste remained lower for both IOB strains than previously reported for e.g., organic acid producing *Aspergillus niger* [29] and cyanide producing *Chromobacterium violaceum* [25].

In this study, the initial cell number at the start of bioleaching was set as 3 × 10^6^ cell·mL^−1^ and the cell numbers remained in the same order of magnitude during the 10-day study. On the contrary, Khaing et al. [14] reported the cell numbers of a strain from *Roseovarius* genus to increase from 3 × 10^6^ cell·mL^−1^ to 5 × 10^8^ cell·mL^−1^ during the bioleaching of gold from sulfide ore. In another study, Khaing et al. [21] reported that initial cell numbers of IOB had no effect on gold dissolution from sulfide ore when the initial cell numbers were 1 × 10^4^ cell·mL^−1^ to 1 × 10^6^ cell·mL^−1^ and during the 10-day incubation the cell numbers increased from 1 × 10^4^ cell·mL^−1^ to 4.2 × 10^7^ cell·mL^−1^. Therefore, the initial cell numbers used in the present study were likely not the limiting factor in the gold bioleaching, but some toxic components of the ore concentrate and e-waste used for bioleaching may have inhibited bacterial growth [30]. Hence, pre-treatment of the materials to remove possible inhibitory compounds should be explored in future studies. Some microbial cells were also detected in spent medium flasks. It may be possible that some native microorganisms were present in the e-waste and ore concentrate, and survived autoclaving as similar cell numbers as observed in spent medium flasks were also found in ore and e-waste control flasks.

The highest concentrations of triiodide during the bioleaching of gold ore concentrate with *R. tolerans* and *R. mucosus* were 406 mg·L^−1^ and 934 mg·L^−1^, respectively and the highest triiodide concentrations with e-waste were 176 mg·L^−1^ and 472 mg·L^−1^ for *R. tolerans* and *R. mucosus*, respectively. These concentrations were notably higher than the concentrations (up to 240 mg·L^−1^) reported by Khaing et al. [14] for other strains of *Roseovarius* genus. During the ore concentrate bioleaching, redox potentials varied from 124 to 350 mV and from 230 to 392 mV depending on the leached material, leaching method and sampling time for *R. tolerans* and *R. mucosus*, respectively. For comparison, the redox potentials reported by Khaing et al. [14,21] during sulfidic ore bioleaching with strains of *Roseovarius* genus were notably higher, 472–547 mV. According to Baghalha [31] AuI_2_^−^ complex is stable at redox potentials from 500 to 700 mV (standard hydrogen electrode), which is approximately 300–500 mV vs. Ag/AgCl. The low redox potentials detected especially during e-waste bioleaching (<300 mV vs. Ag/AgCl) may explain the modest Au leaching yields in the present study. Moreover, the reduction in leaching yield over time in the presence of e-waste indicated the instability of the leached gold under the conditions used. Therefore, future studies should explore the optimization of the bioleaching e.g., by pre-treating the e-waste and ore concentrate with biooxidation to remove oxidant consuming materials before iodide bioleaching.

One advantage of iodide leaching over cyanide leaching is the stability of AuI_2_^−^ complex over a wide pH range of 0–13 [31]. In the present study pH varied from 7.1 to 8.8 and from 7.5 to 9.0 depending on the leached material, leaching method and sampling time for *R. tolerans* and *R. mucosus*, respectively. These were similar to the pH values (pH 7.7–8.8) reported by Khaing et al. [14] during the bioleaching of gold ore with other strains of *Roseovarius* genus. Therefore, pH was unlikely to have a major impact on gold leaching in the present study

In conclusion, this study showed the ability of two culture collection strains, *R. tolerans* DSM 11457^T^ and *R. mucosus* DSM 17069^T^, to bioleach gold from e-waste and sulfidic gold ore concentrate. While the leaching yields from ore concentrate were promising, the yields from e-waste remained low. Limiting factors for bioleaching were likely inhibition of bacterial growth and low redox potential caused by some constituents of the ore concentrate and e-waste used for the bioleaching. Therefore, future studies should explore the pre-treatment of the ore concentrate and e-waste to remove inhibitory and oxidant consuming compounds before bioleaching with IOB to optimize leaching yields. Moreover, the use of IOB could be explored for extracting gold from refractory gold ores to determine whether a prior oxidation step is required before iodine-based gold bioleaching.

## Figures and Tables

**Table 1:** Composition of the gold ore concentrate (ppm).

**Ag**	**Al**	**As**	**Au**	**Ba**	**Be**	**Ca**	**Cd**	**Ce**	**Co**	**Cr**	**Cs**
25	13,400	1420	45	42.6	0.4	11,200	2.93	6.54	570	80	2.2
**Cu**	**Dy**	**Er**	**Eu**	**Fe**	**Ga**	**Gd**	**Hf**	**Hg**	**Ho**	**In**	**K**
2417	0.89	0.68	0.24	442,000	2.93	0.98	1.15	12	0.2	0.13	5260
**La**	**Li**	**Lu**	**Mg**	**Mn**	**Mo**	**Na**	**Nb**	**Nd**	**Ni**	**P**	**Pb**
2.77	6.7	0.12	5160	315	23.7	7420	2.5	4.25	351	1900	41.2
**Pd**	**Pr**	**Pt**	**Rb**	**Re**	**S**	**Sb**	**Se**	**Sm**	**Sn**	**Sr**	**Ta**
<1	0.92	0.484	12.4	0.0269	399,000	80.6	20.2	1.22	1.6	42.1	0.16
**Tb**	**Th**	**Ti**	**Tl**	**Tm**	**U**	**V**	**W**	**Y**	**Yb**	**Zn**	**Zr**
0.14	1.1	6940	1.2	0.09	0.26	157	40.3	4.12	0.59	1020	37

**Table 2:** Composition of e-waste (ppm).

**Ag**	**Al**	**As**	**Au**	**Ba**	**Be**	**Ca**	**Cd**	**Ce**	**Co**	**Cr**	**Cs**
1100	24,000	27.6	1030	10,400	0.3	39,100	0.4	12	21.2	31	0.7
**Cu**	**Dy**	**Er**	**Eu**	**Fe**	**Ga**	**Gd**	**Hf**	**Hg**	**Ho**	**In**	**K**
190,200	20	0.47	4.23	10,100	5.73	3.08	17.9	<0.05	4.99	<0.01	566
**La**	**Li**	**Lu**	**Mg**	**Mn**	**Mo**	**Na**	**Nb**	**Nd**	**Ni**	**P**	**Pb**
11.2	15.2	0.11	1790	475	27.1	799	10.7	38.3	5460	770	1760
**Pd**	**Pr**	**Pt**	**Rb**	**Re**	**S**	**Sb**	**Se**	**Sm**	**Sn**	**Sr**	**Ta**
12	3.24	0.119	0.9	0.0099	841	486	13.4	2.17	36,900	411	23
**Tb**	**Th**	**Ti**	**Tl**	**Tm**	**U**	**V**	**W**	**Y**	**Yb**	**Zn**	**Zr**
0.44	2.96	2600	0.1	<0.05	0.62	13	101	27.1	0.89	456	407

**Table 3:** Two-way analysis of variance (ANOVA) *p*-values to evaluate the significance of the effect of bacterial species and leaching method (excluding uninoculated ore and e-waste controls) on gold leaching from ore concentrate and e-waste after 1, 5 and 10 days. Significant *p*-values < 0.05 are indicated with *.

Material	Parameter	*p*-Values		
		Day 1	Day 5	Day 10
Ore concentrate	Bacterial species	4.12 × 10^−4^ *	0.0226 *	0.197
	Leaching method	0.0115 *	3.21 × 10^−4^ *	0.119
e-waste	Bacterial species	0.0111 *	0.794	0.524
	Leaching method	7.04 × 10^−4^ *	0.0118 *	7.23 × 10^−6^ *

**Table 4:** One-way analysis of variance (ANOVA) *p*-values to evaluate the significance of the effect of leaching method (including uninoculated ore and e-waste controls) on gold leaching from ore concentrate and e-waste after 1, 5 and 10 days. Significant *p*-values < 0.05 are indicated with *.

Material	Parameter	*p*-Values		
		Day 1	Day 5	Day 10
Ore concentrate	*R. tolerans*	0.105	0.00479 *	0.129
	*R. mucosus*	0.0107 *	2.28 × 10^−4^ *	0.0973
e-waste	*R. tolerans*	8.38 × 10^−4^ *	3.48 × 10^−4^ *	0.00721 *
	*R. mucosus*	0.00462 *	0.0347 *	0.0158 *

**Table 5:** Two-way analysis of variance (ANOVA) *p*-values to evaluate the significance of the effect of bacterial species and leaching method (excluding uninoculated ore concentrate and e-waste controls) on triiodide concentrations during gold leaching from ore concentrate and e-waste after 1, 5 and 10 days. Significant *p*-values < 0.05 are indicated with *.

Material	Parameter	*p*-Values		
		Day 1	Day 5	Day 10
Ore concentrate	Bacterial species	2.02 × 10^−6^ *	2.28 × 10^−7^ *	0.999
	Leaching method	1.07 × 10^−8^ *	3.70 × 10^−10^ *	7.54 × 10^−7^ *
e-waste	Bacterial species	4.70 × 10^−4^ *	0.0313 *	9.32 × 10^−3^ *
	Leaching method	0.878	0.574	0.0129 *

**Table 6:** One-way analysis of variance (ANOVA) *p*-values to evaluate the significance of the effect of leaching method (including uninoculated ore and e-waste controls, medium control and bacteria control) on triiodide concentrations during gold leaching from ore concentrate and e-waste after 1, 5 and 10 days. Significant *p*-values < 0.05 are indicated with *.

Material	Parameter	*p*-Values		
		Day 1	Day 5	Day 10
Ore concentrate	*R. tolerans*	4.75 × 10^−5^ *	2.00 × 10^−6^ *	1.12 × 10^−7^ *
	*R. mucosus*	2.77 × 10^−12^ *	1.65 × 10^−13^ *	1.82 × 10^−6^ *
e-waste	*R. tolerans*	3.38 × 10^−12^ *	2.31 × 10^−5^ *	0.0349 *
	*R. mucosus*	3.39 × 10^−9^ *	2.74 × 10^−8^ *	1.21 × 10^−4^ *

**Table 7:** Comparison of gold bioleaching with various microbes and experimental conditions.

Microorganism	Gold Source (Pulp Density)	Nutrient	Leaching (%)	Temperature (^°^C)	Time (d)	pH	Initial Cell Number (Cells·mL^−1^)	Reference
***R. tolerans***	Sulfide ore concentrate (1%)	MB + KI ^1^	100	30	10	7–8	3 × 10^6^	This study
***R. mucosus***	Sulfide ore concentrate (1%)	MB + KI ^1^	34	30	10	8	3 × 10^6^	This study
***R. tolerans***	PCBs (1%)	MB + KI ^1^	1.0	30	10	7–8	3 × 10^6^	This study
***R. mucosus***	PCBs (1%)	MB + KI ^1^	1.0	30	10	7–8	3 × 10^6^	This study
***R. tolerans***	Sulfide ore (3.3%)	MB + KI ^1^	100	30–35	10	7.7–8.4	1 × 10^4^−3 × 10^6^	[14,21]
***Aspergillus niger***	PCBs (1.3%)	G+ ^2^	87	28	14	4.4–6.6	-	[29]
***Chromobacterium violaceum***	PCBs (1.5%)	YP ^3^	10.8	30	8	11	-	[25]

^1^ MB + KI = Marine broth (18.7 g·L^−1^), KI (10.9 g·L^−1^). ^2^ G+ = glucose (50 g·L^−1^), CaCl_2_ (0.1 g·L^−1^), NH_4_Cl (1.5 g·L^−1^), MgSO_4_·7H_2_O (0.025 g·L^−1^) and KH_2_PO_4_ (0.5 g·L^−1^), pH 4.4. ^3^ YP = yeast extract (5 g·L^−1^), polypeptone (10 g·L^−1^), glycine (5 g·L^−1^), MgSO_4_·7H_2_O (1 g·L^−1^).
